# Mechanism of UV-C-Induced Oxygen Vacancies Altering the Colour of Dental Zirconia

**DOI:** 10.3390/ma19071427

**Published:** 2026-04-02

**Authors:** Mengxiao Xu, Xuedong Bai, Siyu Yang, Weijia Wen, Kiho Cho, Yun-Hong Lee, Shixin Jin, James Kit Hon Tsoi

**Affiliations:** 1 Dental Materials Science, Applied Oral Sciences and Community Dental Care, Faculty of Dentistry, The University of Hong Kong, Hong Kong SAR, China; mercyx@connect.hku.hk (M.X.); weissbxd@connect.hku.hk (X.B.); dkcho@hku.hk (K.C.); ralyhong@hku.hk (Y.-H.L.); jkhtsoi@hku.hk (J.K.H.T.); 2 Department of Physics, The Hong Kong University of Science and Technology, Hong Kong SAR, China; phsidney@ust.hk; 3 Function Hub, The Hong Kong University of Science and Technology (Guangzhou), Guangzhou 511453, China; phwen@ust.hk

**Keywords:** oxygen vacancy, defect engineering, zirconia, band gap, ultraviolet (UV)

## Abstract

UV-C irradiation enables digital zirconia colouring. This study investigates the atomic mechanism driving this defect-induced optical change. The band gap was calculated from the absorption spectra with the Tauc plot. The absorption spectra were measured using UV–visible spectroscopy. The surface composition was evaluated through X-ray photoelectron spectroscopy (XPS). The location of the oxygen vacancy was tested through electron paramagnetic resonance (EPR). The computer calculation using Density Functional Theory was conducted and the density of states (DOSs) were calculated. The band gap reduced rapidly from the baseline group (3.184 eV) to the 30 min irradiated group (3.097 eV). The XPS results showed that the electron density around O1s reduced and the electron density around Zr 3d increased. The EPR signal (g = 2.0037) increases progressively as the UV-C irradiation time is prolonged from 15 min to 24 h, indicating the accumulation of paramagnetic defect centres. The DOSs suggested the emergence of defect-associated states and band-edge tailing in oxygen deficient models, consistent with the experimentally observed reduction in the Tauc-derived optical band gap. This study confirmed the mechanism by which UV-C-induced oxygen vacancies modify the colour of 3Y-TZP.

## 1. Introduction

Defects in materials are often considered detrimental, potentially decreasing mechanical properties or altering optical properties. However, controlling defects is necessary to modulate the electrical, magnetic, and chemical properties of materials used in solar cells, magnets, and catalysts [1]. Defect engineering involves creating and controlling defects in a material’s microstructure to tailor its properties. Defects determine some of the properties of crystalline materials. In dentistry, zirconia is commonly used as a restorative material due to its optimal mechanical, biological, and esthetic properties [2]. Although its white colour is similar to that of some natural teeth, manual post-staining processing is still needed to adjust the restoration’s colour and translucency to match an individual’s teeth. Bai et al. proposed a new defect engineering method to digitally tailor the colour of dental zirconia, using UV-C irradiation to create oxygen vacancies and thereby alter the colour [3]. To sophisticatedly manipulate the colour of dental zirconia with UV-C irradiation in the future, we studied the mechanisms of defect generation and optical property alternation in atom-scale through experimental and theoretical methods.

**Figure 1:**
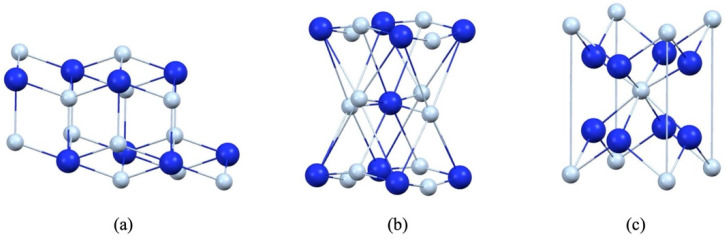
Crystal structure of zirconia: (**a**) monoclinic structure; (**b**) tetragonal structure; (**c**) cubic structure; Zr in dark blue and O in light blue.

In addition to being widely employed as a thermal insulation material, zirconium dioxide (zirconia, ZrO_2_) has been extensively employed as a dental restoration and implant material since the 2000s [2,4]. Zirconia has three primary crystalline structures, monoclinic, tetragonal, and cubic structures, as shown in Figure 1. Dental zirconia ceramics are categorized into three generation: the first is 3 mol% yttria-stabilized tetragonal zirconia polycrystals (3Y-TZPs); the second retains 3Y-TZPs but added colourants for improved shading; the third is 4–5 mol% yttria-stabilized tetragonal zirconia polycrystals (4-5Y-TZPs) with an addition of 10–50 wt.% cubic phase for superior translucency. The third generation is the most popular one in dentistry because of its proximal translucency, which resembles natural teeth. However, adding cubic phase content decreases the mechanical capacity, such as strength and fracture toughness. With 50% cubic phase added, 5Y-TZP exhibits only comparable strength and fracture toughness to lithia silicate glass–ceramics employed in indirect dental restorations [5].

Beyond mechanical properties, colour and translucency are essential esthetic properties of dental zirconia. Pure zirconia appears opaque and white, while dental zirconia requires certain translucency and a yellowish colour to mimic natural human teeth. The translucency of a zirconia prosthesis is achieved by introducing the cubic phase to 3-5 Y-TZ, while colour is modified by colourant additions [6]. However, to accommodate individual patients’ conditions, post-treatment is usually needed. This involves manual staining and glazing a porcelain layer for a tooth-like appearance. This work is complex and requires high artistic skills. The additional layer of porcelain also requires more tooth substance reduction. Furthermore, the porcelain layer is prone to chipping and delamination, leading to a decrease in mechanical properties.

Therefore, new methods are needed to modify the colour of dental zirconia without glazing, replacing the manual work with a digital process. In our hypothesis, when irradiated with UV-C light, the zirconia surface absorbs photon energy. From the energy scale, the electrons are excited to nearby orbitals due to the absorbed energy exceeding a threshold [7]; from quantum theory, a mid-gap state in the forbidden gap is formed due to the electrons. At the atomic and electronic scales, UV-C irradiation may induce oxygen-related defect states through photoinduced electronic and surface chemical processes [8]. This defect-induced modification of the optical band gap modulates the esthetic properties of dental zirconia without reducing mechanical properties [3].

However, no studies have combined experimental characterization and theoretical simulation to investigate the mechanism underlying the intrinsic change in dental zirconia. Here, we characterized oxygen vacancies with EPR and XPS, band gaps with UV-vis, and performed DFT calculations, simulating the density of states in different oxygen vacancy concentrations to compare with the experimental change.

## 2. Materials and Methods

### 2.1. Materials

The 3 mol% yttria-stabilized tetragonal zirconia polycrystal (Upecra ST, Shenzhen Upcera Dental Technology, Shenzhen, China) was used in this study. The samples were cut to the required sizes according to different tests. Discs with 15 mm diameter and 1.2 mm thickness were used for UV-vis spectroscopy. Chips with 3 mm length and width, and 1 mm thickness were employed for the X-ray photoelectron spectroscopy (XPS) and electron paramagnetic resonance (EPR). The samples were irradiated with 222 nm UV-C light for 15 min, 30 min, 1 h, 4 h, and 24 h. The samples were placed on a metal platform 2 cm under the UV-C light at room temperature (20~25 °C) in air. The irradiance of the UV-C light was 1.870 mW/cm^2^, tested with an irradiance meter (HPL-220UV-C, HopooColor Technology, Hangzhou, China). The groups in the following figures are labelled as 15, 30, 60, 240, and 1440. The doses (Gy) of the disc samples under 15, 30, 60, 240, and 1440 min of irradiation are 1.202 Gy, 2.404 Gy, 4.808 Gy, 19.234 Gy, and 115.404 Gy; the doses (Gy) of the chip samples under 15, 30, 60, 240, and 1440 min of irradiation are 28.050 Gy, 56.106 Gy, 112.212 Gy, 448.848 Gy, and 2693.088 Gy.

### 2.2. Characterization Methods

The surface chemical composition was analyzed by X-ray photoelectron spectroscopy (XPS, Thermofisher Nexsa, Thermo Fisher Scientific, Hilldboro, OR, USA), with an anode source of AlKa (150 W). The binding energies of C 1s, O 1s, and Zr 3d were tested for one sample from every group. Then, every spectrum was deconvoluted into isolated peaks. The software Thermo Avantage 5.9931 (Avantage Data System, Thermo Fisher Scientific, Hilldboro, OR, USA) was used to analyze XPS data. Electron paramagnetic resonance (EMXplus-9.5/12, Bruker BioSpin, Ettlingen, Germany) was performed to confirm the presence of unpaired electrons in zirconia at each irradiation time at room temperature (20~25 °C). The X-band frequency was 9.848 GHz; the centre field was 3500 g; the modulation amplitude was 1 G; the microwave power was 20 mW; the scanning width was 100 G; and the scanning time was 5 min for one sample. The band gap was determined from Tauc plots derived from the absorption spectra. UV–visible spectroscopy was performed to obtain absorption spectra from 200 nm to 800 nm using a Lambda 1050+ spectrometer (PerkinElmer, PerkinElmer Inc., ChemDraw, Waltham, MA, USA).

### 2.3. Computational Calculation

For computational calculations, the CASTEP module in Materials Studio 2024 (BioVia, Dassault Systèmes, San Diego, CA, USA) was used to calculate the density of states (DOSs) using the Density Functional Theory (DFT) method. The crystal structure was built according to the zirconia tetragonal structure (No. 1526427) from the Cambridge Crystallographic Data Centre (The Cambridge Crystallographic Data Centre Ltd., Cambridge, UK) using Mercury 2021.3.0 (The Cambridge Crystallographic Data Centre Ltd., Cambridge, UK). The structure used for simulation was a supercell with dimensions of 2 × 2 × 2 built from No. 1526427. One yttrium atom replaced a zirconium atom, representing a 3 mol% T-TZP. The geometry optimization and the energy calculation were conducted with GGA-PBE, the Perdew–Burke–Ernzerhof (PBE) functional in the Generalized Gradient Approximation (GGA) method. The cut-off energy was set to 500 eV, and the k-point grid was 3 × 3 × 2. After geometry optimizations, the oxygen atoms were deleted according to the representative oxygen vacancy concentrations. The charge was set to be zero for no oxygen vacancy, adding −2 for one oxygen vacancy. The density of states was visualized by Materials Studio 2024.

## 3. Results

### 3.1. Colour Change Observations

Based on the previous study by Bai et al., the colour difference of 3Y-TZP increased logarithmically with UV-C irradiation time [3]. Longer exposure produced more intense yellowish shades, while translucency decreased slightly as the colour became darker. The surface morphology and mechanical strength remained unchanged after the irradiation process.

### 3.2. Surface Composition (XPS)

**Figure 2:**
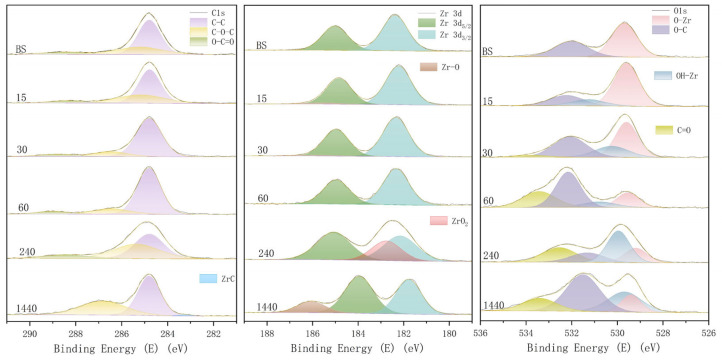
XPS high-resolution spectra of C 1s, Zr 3d, and O 1s.

The full XPS spectra confirmed the presence of C 1s, O 1s, Zr 3d, and Y 3d signals. The high-resolution spectra revealed specific shifts in binding energy, as shown in Figure 2. The charge of high-resolution spectra was corrected according to the standard C-C binding energy of 284.8 eV.

The main peak shifted to lower binding energy from 182.6 eV (Baseline/BS) to 181.9 eV (15 min), 182.01 eV (30 min), 182 eV (60 min), 181.84 eV (4 h), and 181.78 eV (24 h). In the 4 h (240 min) group, the Full Width at Half Maximum (FWHM) of the main peak widened significantly. In the 24 h (1440 min) group, an increase in sub-stoichiometric Zr-O was observed. The metal oxide (O-Zr) peak at 529.29 eV (BS) shifted to higher energy in irradiated groups. The atomic percentage of O-Zr decreased notably from BS to 24 h. Conversely, the OH-Zr component increased from BS to 4 h and 24 h. In the 4 h and 24 h groups, multiple splitting appeared in the lattice peak, correlating with an increase in OH-Zr, O-C, and O=C percentages. The C-C peak atomic ratio was high in the BS and short-irradiation groups (15–60 min). However, the C-O-C atomic ratio increased significantly in the 4 h (43.16%) and 24 h (41.45%) groups compared to the BS group (28.52%).

### 3.3. Defect Location (EPR)

**Figure 3:**
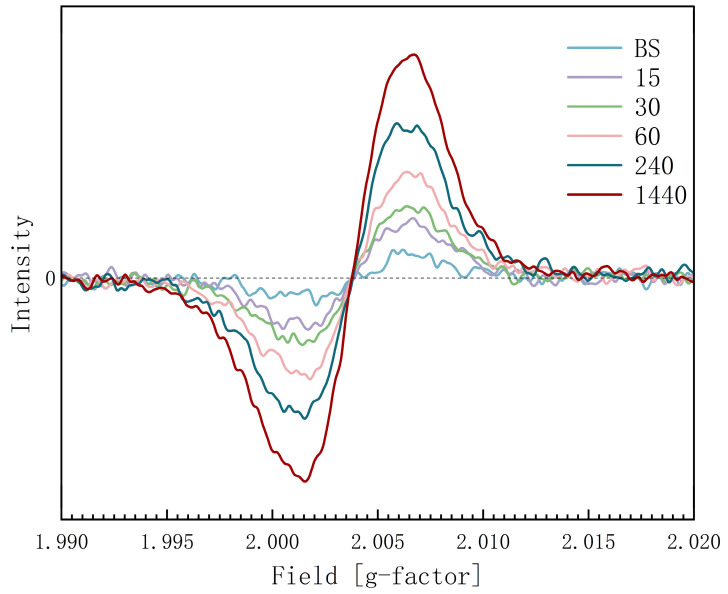
EPR signals.

The EPR signals are shown in Figure 3. The baseline group showed a low signal, indicating few evident paramagnetic defects. All UV-C-irradiated groups exhibited a significantly elevated signal centred at a g-factor of approximately 2.00374. The signal intensity demonstrated a clear, positive correlation with the duration of UV-C irradiation, increasing progressively from 15 min to 24 h.

### 3.4. Band Gap and DOS Simulation

**Figure 4:**
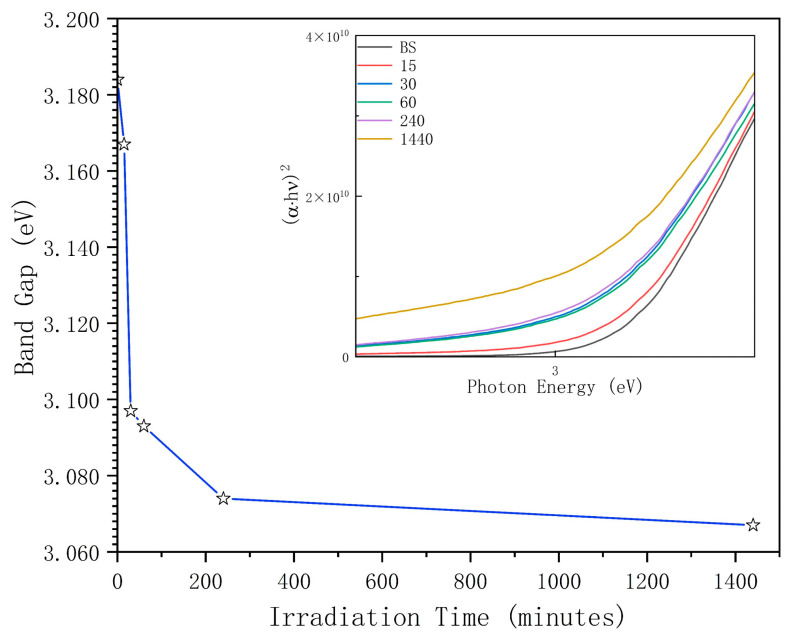
Band gap–irradiation time plot with the Tauc plot in the corner.

The band gap, calculated from the Tauc plot according to the absorption spectrum and Equation (1), is shown in Figure 4, with the Tauc plots in the corner. In Equation (1), *α* is the absorption coefficient obtained by normalizing absorption data corresponding to path length; *h* is the Planck constant; ν is the frequency of incident photons; *B* is a constant; and *E_g_* is the band gap energy. The value *γ* is 12 due to the direct band gap of tetragonal zirconia [9].

(1) $${\left(\alpha \cdot h\nu \right)}^{1/\gamma }=B(h\nu -E_{g})$$

The baseline group displayed an initial band gap energy of 3.184 eV. After irradiation, the band gap reduced to 3.097 eV within the first 30 min and eventually stabilized at 3.067 eV after 24 h (1440 min). The change around 0.110 eV might come from a system error, but, among six samples with variant irradiation times, the optical band gap decreases with prolonged UV-C irradiation time.

**Figure 5:**
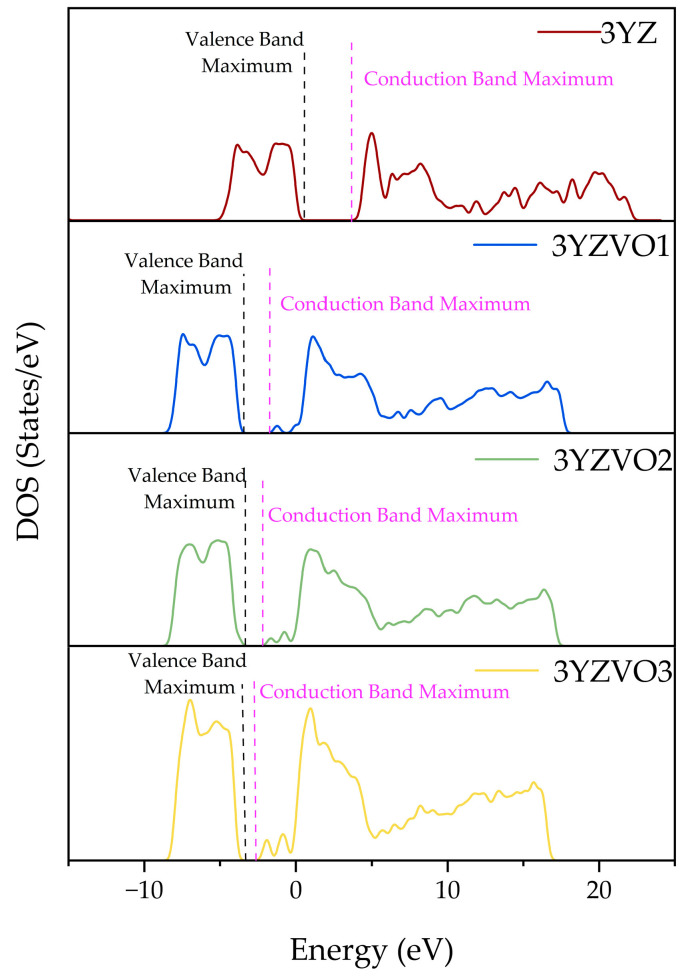
Simulated DOSs of structures of 3YZ, 3YZVO1, 3YZVO2, and 3YZVO3.

The density of states (DOSs) simulation (Figure 5) for the standard 3YZ structure exhibited a clean forbidden gap. The structure with one oxygen vacancy (3YZVO1) introduced a localized energy state below the conduction gap. As the vacancy concentration increased (3YZVO2 and 3YZVO3), the density of these mid-gap states increased significantly.

## 4. Discussion

### 4.1. Interpretation of Colour and Feasibility

The logarithmic increase in yellowish shading without compromising mechanical strength or surface morphology confirms the feasibility of using 222 nm UV-C irradiation as a defect engineering method for digital shade matching in dental zirconia [3].

### 4.2. Analysis of Surface Chemical Changes

XPS provides outermost surface chemical evidence consistent with oxygen-deficient zirconium environments. The shift to lower binding energy indicates an increase in electron cloud density around Zr atoms. The multiple splitting observed in the 4 h group suggests the presence of ZrO_x_ (0 < x < 2) [10], and the increase in sub-stoichiometric Zr-O after 1440 min indicates enhanced Zr-O interactions [11]. The shift in the O-Zr peak to higher energy reflects a decrease in electron cloud density [12]. The dramatic reduction in metal oxide (O-Zr) combines with the rise in OH-Zr from BS to 1440 min. In addition, the appearance of lower-coordination oxygen states (O-C, O=C) further supports that the redistribution of O 1s components [13]. The increase in C-O-C ratios suggests that oxygen vacancies with electron holes act as active sites, promoting the photocatalytic degradation of surface carbon contaminants [14]. Short-term irradiation may partially modify or oxidize adventitious surface carbon species [15].

### 4.3. Confirmation of Paramagnetic Defects

The EPR signal centred at g ≈ 2.00374 is close to the free-electron g-factor (g_e_ = 2.0023), which is a classic signature of paramagnetic oxygen-related defects [16]. The progressive increase in signal intensity proves that UV-C irradiation continuously generates and accumulates these paramagnetic centres [17,18]. The progressive increase in signal intensity from 15 min to 24 h demonstrates that the concentration of the paramagnetic defects is UV-C-dose-dependent. This indicates that prolonged UV-C exposure leads to a continuous accumulation of oxygen vacancies within the microstructure of the 3Y-TZP, which can significantly modulate its electronic and chemical properties. Although, the penetration depth of UV-C light is shallow, so the generation of defects might concentrate on the outmost surface. The results of EPR testing with the whole 3 × 3 × 1 mm sample still can depict the generation and the enhancement of the paramagnetic defects induced by UV-C irradiation.

### 4.4. Proposed Mechanism of UV-C-Induced Colour Change

Based on the combined experimental and computational findings, a defect-mediated mechanism can be proposed for the UV-C-induced colour alteration of 3Y-TZPs [19,20]. These defect-related changes affect the optical behaviour of zirconia by introducing and enhancing localized electronic states that participate in light absorption. High-energy photons (5.2 eV) from UV-C light excite electrons from the valence band [21,22,23], forming a localized energy state below the conduction band in the presence of oxygen vacancies [20]. The simulation of higher vacancy concentrations (representing prolonged irradiation) shows the extended localized energy state below the conduction band with a reduced electronic band gap [24,25]. Consequently, the experimental and theoretical results converge to confirm the decrease in the band gap, leading to the modification of the optical absorption, which ultimately results in a visible colour change.

Under 222 nm irradiation, the zirconia surface experiences photoinduced electronic excitation that promotes changes in the near-surface chemical and electronic environments. These changes are reflected in the XPS spectra as altered oxygen and zirconium bonding states, and in the EPR spectra as the accumulation of paramagnetic defect centres.

## 5. Conclusions

This study elucidates the atomic-scale mechanism of direct colour printing on 3Y-TZP using 222 nm UV-C irradiation. Experimental analysis via XPS and EPR confirmed that high-energy photons induce the formation of singly ionized oxygen vacancies, with the defect concentration increasing proportionally to the irradiation duration. This accumulation of intrinsic defects reduces the optical band gap, a phenomenon corroborated by DFT simulations that reveal the formation of mid-gap states within the forbidden gap. Consequently, the visible colour alteration is directly attributed to the modification of the electronic structure caused by these photon-induced oxygen vacancies. These findings validate defect engineering as a precise mechanism for digital shade matching in dental zirconia, offering a controllable and non-destructive alternative to traditional manual staining methods.

## Data Availability

The data presented in this study are available on request from the corresponding author.
